# Knowledge, Protective Behaviors and Risk Perception of COVID-19 among Dental Students in India: A Cross-Sectional Analysis

**DOI:** 10.3390/healthcare9050574

**Published:** 2021-05-13

**Authors:** Kavita Batra, Yashashri Urankar, Ravi Batra, Aaron F. Gomes, Meru S, Pragati Kaurani

**Affiliations:** 1 Office of Research, School of Medicine, University of Nevada, Las Vegas, NV 89102, USA; 2 Community Health Centers of South-Central Texas, Gonzales, TX 78629, USA; UrankarY@chcsct.com; 3 Department of Information Technology, Coforge Ltd., Atlanta, GA 30338, USA; ravi.batra@coforgetech.com; 4 Department of Periodontology and Oral Implantology, Uttaranchal Dental and Medical Research Institute, Dehradun 248140, India; aarongomes@hotmail.com; 5 Department of Oral Medicine and Radiology, Uttaranchal Dental and Medical Research Institute, Dehradun 248140, India; drmeru@gmail.com; 6 Department of Prosthodontics, Mahatma Gandhi Dental College and Hospital, Jaipur 302022, India; drpragatikaurani@rediffmail.com

**Keywords:** COVID-19, dental precautions, dental students, India, infection control, knowledge, perception, survey

## Abstract

Objective: This study’s objective was to examine the knowledge, performance in practicing protective behaviors, and risk perception of Coronavirus disease-19 (COVID-19) among dental students of India. Methods: A web-based cross-sectional survey was conducted from 10–30 August 2020, involving 381 dental students that were enrolled at the Uttaranchal Dental and Medical Research Institute in India. A web-based structured questionnaire assessed the COVID-19 related knowledge, protective behaviors, and risk perception performance. The independent-samples-t and analysis of variance tests were used to analyze the differences in knowledge, protective behaviors, and perception across the groups. Results: Of the dental students surveyed, 83% had adequate knowledge of COVID-19, and nearly 80% followed appropriate practices regarding COVID-19. The COVID-19 related risk perception was higher among females as compared to males. COVID-19 related knowledge was significantly correlated with preventive behaviors (*r* = 0.18; *p* < 0·01) and risk perception (*r* = 0.10; *p* < 0.05). We found a high score of COVID-19 related knowledge and precautionary behaviors and moderate risk perception among students. Conclusions: Knowledge and protective behaviors towards infectious diseases, such as COVID-19, have clinical applications in developing educational and formal training programs to promote adherence to the infection control practices among dental students. Clinical significance: The findings of this study will inform policymakers to emphasize on effective risk communication. Dental institutions can incorporate infection control modules in the current curriculum, thereby making future dental professionals capable of performing effective infection control management in the clinical settings. This is critical in improving their knowledge of infection control practices to minimize the risk of nosocomial infections.

## 1. Introduction

The World Health Organization (WHO), on 11 March 2020, declared novel Coronavirus disease (COVID-19) a pandemic caused by a viral strain, named as SARS-CoV-2 [1]. There are more than 104,370,550 confirmed cases globally, and 2,271,180 deaths were reported as of 5 February 2021, while writing this manuscript [1]. Currently, there are nearly 10,814,656 confirmed COVID-19 cases in India alone [2]. COVID-19 reported to be highly contagious when compared to its previous predecessors [3], and it possesses a relatively higher reproductive number (R_0_), ranging from 2 to 4. In other words, on an average, a COVID-19 infected individual can transmit the virus or infect 2–4 individuals during the course of time [3]. The primary mode of transmission of COVID-19 is person-to-person via respiratory droplets either inhaled or through contact, with the typical incubation period ranging from 2–14 days [4,5,6]. Globally, regulatory bodies provided infection control guidelines and standard operating procedures to curb the COVID-19 transmission in the dental settings [7]. These guidelines were related to clinical workspace, the use of personal protective equipment (PPE), disposal of PPE, disinfection of surfaces, and operatory to minimize the COVID-19 transmission. In March, 2020, the Dental Council of India (DCI) issued its first set of advisory, which were later updated during the course of pandemic [8].

To minimize the transmission of the virus, public health organizations have recommended standard preventive measures, including the use of face coverings, practicing social distancing, maintaining hand hygiene, and limiting contact with infected individuals [9,10]. These public health actions are necessary to minimize the spread; however, challenges that are associated with behavior adoption can pose significant barriers [11]. Furthermore, the dissemination of a vast amount of unreliable and unclear information on social media can complicate containment measures of COVID-19 and induce panic among the public [11,12,13].

Dental professionals and dental students are especially vulnerable due to their proximity to symptomatic or asymptomatic patients and their oral fluids [14,15]. According to one Japan-based study, the SARS-CoV-2 virus was found in 11 out of 12 COVID-19 patients [15]. The aerosols and droplets that were generated through dental procedures, the possible inhalation of airborne microorganisms, and direct or indirect contact with contaminated instruments are the potential routes of transmission in the dental care settings [16,17,18]. Previous studies that were conducted in Iran, Peru, and India reported insufficient knowledge of infection control practices among dental professionals [19,20,21,22]. The adherence to protective behaviors among healthcare students is enormously crucial among countries with healthcare workforce scarcity [23]. According to the National Health Profile, India has one doctor per 10,000 population of patients instead of the WHO recommendation of having one doctor per 1000 population of patients [11]. The apparent deficiency in the number of healthcare workforce can be potentially offset by dental workforce [23]. India achieved a higher dentist-to-patient ratio of 1:5000 as compared to WHO recommended ratio of 1:7500, with the rural–urban distribution being uneven [11]. In times of a healthcare crisis, dental professionals/students can be assigned hospital-based responsibilities to assist in the response efforts and gain practical experience to handle such future outbreaks [23]. In this context, knowledge regarding the prevention and control of COVID-19 transmission is essential for dental professionals/students to improve the preparedness and response actions. Informing future dentists about a pandemic disease at the initial learning stage is critical. Thus, they will be better equipped to play an active role in preventing and controlling disease during future outbreaks or spikes in the ongoing pandemic. Therefore, this study aimed to assess the knowledge, risk perception, and adherence to preventive behaviors among dental students regarding the COVID-19 pandemic.

## 2. Materials and Methods

### 2.1. Study Design and Study Participants

This descriptive, cross-sectional survey study was conducted from 10 August 2020 to 30 August 2020, among dental students in India. Full-time dental students (undergraduate, interns, and postgraduate), currently enrolled in any of the private, government, or deemed universities, who were of Indian nationality, were aged 18 years or above, had internet access, could provide informed consent, and comprehend English, were invited to participate in this survey. Part-time students, alumni, and those with no internet access and an inability to understand English were excluded from this study.

### 2.2. Ethical Consideration

The ethical approval was obtained from the institutional ethics committee (Uttaranchal Dental and Medical Research Institute [UDMRI], Mazri Grant, Dehradun, India., Ref. No. IEC/PA-02/2020, 8 August 2020). The ethics committee reviewed and approved this study protocol, participant information sheet (PIS), informed consent form, and the survey questionnaire. All of the study participants were requested to sign the informed consent to confirm their willingness to participate by answering an agree/disagree question. Informed consent included detailed information that was related to the aim and significance of the study so that participants could make an informed choice about whether to participate or withdraw at any time if he/she so wished. The participants who selected the “agree” option were directed to complete the self-administered questionnaire. All of the participants’ anonymity was ensured, and no personal identifiers, including names, email IDs, and details of COVID-19 exposure, were collected. Only one response per Internet Protocol (IP) address was allowed.

### 2.3. Recruitment and Data Collection

This web-based survey was developed through Qualtrics (Provo, UT). The convenience sampling method was used for sample recruitment. The survey link was disseminated through emails and WhatsApp groups among the target student populations. The WhatsApp groups was otherwise used by faculty members to deliver notes, announcements, and lectures as part of a virtual instruction model that was recently implemented in response to the COVID-19 pandemic. The questionnaire was sent to 480 students, and 381 completed the survey. Therefore, the response rate was 80.2%.

### 2.4. Survey Instruments and Variables

The online survey questionnaire had a total of 33 questions and four sections: (1) demographic information; (2) COVID-19 related knowledge; (3) questions that were related to preventive behaviors for COVID-19; and, (4) questions to assess risk perception of COVID-19.

Demographics: the first section of the questionnaire contained six questions related to demographics, including gender, age, degree course, and COVID-19 education. These questions were developed based on previous literature [24,25].

COVID-19 related knowledge: we used 15 items questionnaire for assessing COVID-19 related knowledge. This tool has been validated by previous studies during historical outbreaks, such as Middle East Respiratory Syndrome (MERS) and Severe Acute Respiratory Syndrome (SARS), and has the reliability (Cronbach’s α) of 0.72 with a content validity index of 0.95 [25,26]. This tool includes questions on the COVID-19 causes (three items); modes of transmission (two items); symptoms and latent period (two items); prevention, assessment, and treatment methods (seven items); and, guidelines for patient care (one item) [25,27]. These questions had possible responses of true/false/not sure options [25,26,27]. A correct answer was assigned one point, and an incorrect/unknown answer was assigned 0 points [25,26,27]. The total knowledge score ranged from 0 to 15, with a higher score [25,26,27] indicating a better knowledge of COVID-19.

Preventive behaviors for COVID-19: this tool was used to assess the performance rate for COVID-19-related preventive behaviors among participants. This tool has been previously used and validated by previous studies [25] on MERS. There was a total of nine items, of which five items were related to minimizing the use of public spaces, avoiding social gatherings, outdoor activities, and enclosed spaces, and minimizing contact with people showing cough symptoms, three items about practicing good hygiene, including handwashing, cleaning, and sanitization, and one item about talking with people about measures to take after COVID-19 infection [25,27]. The behavior (if practiced) was assigned 1 point, and 0 points were assigned if a behavior was not practiced by the participant. The total score ranged from 0–9; a high score was indicative of a high-performance rate [25,27]. The questionnaire has been validated and has a reliability index (Cronbach’s α) of 0.77 [24,25,26,27].

Risk perception of COVID-19: COVID-19 related risk perception was conceptualized as the participant’s fear of being infected with COVID-19. The scale of risk perception [25,26,27] had two items (i.e., ‘I may be infected with COVID-19 more easily than others’ and ‘I am afraid to be infected with COVID-19’). Each item was assessed on a five-point scale from 1 (Never) to 5 (Always). The scale has been validated by previous studies [25,26,27], which were performed during SARS [26] and MERS [27] outbreaks.

### 2.5. Statistical Analysis

Participants’ responses, from Qualtrics, were exported to Microsoft Excel (Microsoft Corporation, Richmond, WV, USA), and then imported to IBM SPSS version 26.0 (IBM Corp. Armonk, NY, USA). COVID-19 related knowledge, preventive practices, and risk perception were the quantitative variables in this study. Descriptive statistics, including the frequencies, proportions, mean, and standard deviations, were generated. Independent-samples- *t*-test and analysis of variance were utilized to analyze the differences in mean scores across different students’ groups. The Pearson’s correlations test was utilized to calculate the correlations among the variables. *p*-values less than 0.05 (two-sided) were considered to be statistically significant, and data were reported as 95% confidence intervals. The priori power analysis was conducted through G power (version 3.1) to ascertain the required sample size for a test with a predetermined alpha, beta (power), and Cohen’s effect size conventions [28,29]. The sample size of n = 302 was considered to be appropriate after factoring in 20% incomplete entries or missing values.

## 3. Results

### Sample Characteristics

A total of 381 responses were recorded during the survey period. The average time that was taken to complete the survey was 8.4 min. The demographic profile of the respondents shows that 268 (70.3%) respondents were females, and 79 (20.7%) were males (Table 1). The average age of the sample was 22.8 years (SD = 2.8 years). The majority of the participants were undergraduates (80%, n = 320), with nearly 48% (154 out of 320) being in the third and fourth year of the undergraduate dental program (Table 1). Approximately 64.8% participants had received some form of COVID-19 related education, and 50.7% of the COVID-19 related information was received from social media (Table 1). There were significant gender differences in the mean scores of knowledge and risk perception (*p* < 0.05). The mean knowledge scores were higher among male participants (M = 13.91, SD = 0.78) than females (M = 13.66, SD = 0.9), with a statistically significant mean difference, M = 0.25 95% CI [0.028, 0.46], *p* = 0.04, Table 1). Significant differences in the risk perception across gender were also noted (*p* = 0.01, Table 1). The mean score of risk perception was slightly higher (M = 3.0, SD = 1.0) among participants without prior COVID-19 education when compared to those who had some sort of education (M = 2.7, SD = 1.0), with a statistically significant mean difference, M = −0.30, 95% CI [−0.05, −0.55], *p* = 0.02, Table 1). There were no statistically significant differences found in the mean scores of preventive behaviors across any sample categories. The mean level of COVID-19-related knowledge was 83.0%. The COVID-19 knowledge item with the highest correct-answer rate (91.3%) was ‘The first case of COVID-19 was diagnosed in Wuhan, China. (T)’. However, the items with the lowest correct answer rates were ‘The disease can be treated by usual antiviral drugs. (F)’ (45.4%) and ‘Only during intubation, suction, bronchoscopy, and cardiopulmonary resuscitation, you have to wear an N95 mask (T)’ (50.4%). The mean performance rate of COVID-related preventive behaviors was 79.9%. The behavior items with the highest performance rates were ‘I reduced the use of public transportation’ and ‘I went shopping less frequently’ and ‘I reduced the use of closed spaces, such as library and theatre’ (90.8%, Table 2). However, the items with relatively lower performance rates were ‘I discussed, with my family and friends, what we should do if infected with COVID-19’ (82.9%) and ‘I increased the frequency of cleaning and disinfecting items that can be easily touched with hands (i.e., door handles and surfaces)’ (86.3%). The total mean score of COVID-19 related risk perception was 2·78 out of 5, and the score for fear of being infected with COVID-19 was 3.1 out of 5 (Table 3). COVID-19 related knowledge was significantly correlated with preventive behaviors (*r* = 0.18; *p* < 0.01) and risk perception (*r* = 0.10; *p* < 0.05). Besides, risk perception was significantly correlated with age (*r* = 0.27; *p* < 0.01; Table 4).

## 4. Discussion

The current descriptive study assessed the knowledge, risk perception, and adherence to the protective behaviors of dental students in India regarding COVID-19. We found moderate levels of COVID-19 related knowledge (83.0%) and adherence to protective behaviors (79.9%) among the dental students. In our sample, nearly half (50.7%) of the study participants obtained COVID-19 education through social media, and only 9.4% reported obtaining the knowledge from college. These results were consistent with another Nigeria based study of undergraduate dental students [18]. Every three out of four students reported lack of formal COVID-19 related education or training in the college settings, as reported by a Turkish study [30]. This might be due to the lack of time available to the universities or colleges to design education programs focused on transmission-based infection control practices following the sudden invasion by the COVID-19 pandemic [11,16,25]. Transmission-based precautions differ from standard measures in providing additional guidance in controlling the spread of rapidly evolving pathogens, such as Coronavirus [31,32,33]. In addition, previous studies found that dental education in India emphasizes blood-borne infection control practices with limited training on the prevention and control of airborne or droplet infections [16]. The dental council of India published comprehensive infection control guidelines in 2009, which seem to be underutilized, even after a decade now [32]. In 2011, knowledge, attitudes, and practices of dental safety among 1874 dentists across eight countries were assessed and only 50% of participants reported utilizing standard infection control practices [33]. Therefore, this study underscores the need to revise the dental curriculum for developing a comprehensive module to teach effective transmission-based infection control practices to the students.

The study item with the highest scores of 91.3% was about the knowledge related to the origin of the COVID-19 pandemic. However, one item (i.e., ‘disease could be treated by common antiviral drugs’) had the lowest correct answer rates of 45.4%. This may be attributed to the rapidly evolving information that was related to the treatment of COVID-19 and surrounding controversies [34]. In this study, an 88.7% correct answer rate was found with the question that was related to the incubation period, which is greater than that reported in a Jordan based research (36.1%). This disparity may be due to the study period’s difference; the Jordanian study was conducted in the early phases of the pandemic when epidemiological characteristics of the COVID-19 were not yet unfolded [35]. Consistent with previous reports [18,35,36], our study participants had a good knowledge of COVID-19 symptoms, which is essential for the early detection of suspected cases to take prompt actions. The performance rate to adhere to the protective behaviors was 79.9%, which was slightly higher than the 73.8% reported by a study conducted in China [37].

Inconsistent with a Nigeria-based study, our study found variations in the risk perceptions by gender [38]. The mean scores for risk perception about the COVID-19 were higher among female students than males (2.90 vs. 2.48, *p* = 0.01). The higher risk perception among females can be explained by their unrealistic perception, gender socialization, and awareness of health warnings [39,40,41]. Additionally, gender also predicted adherence to the protective behaviors, suggesting that females had a higher score of protective behaviors than males. These findings were consistent with the previous study [38]. The knowledge scores of females were lower than the males, and this finding was inconsistent with the other studies [37,42]. Our study found a higher score among postgraduate students than undergraduates, because clinical students rely more on science-based resources, such as the Ministry of Health rather than social media [43]. There were significant differences found in the COVID-19 knowledge and protective behaviors among clinical and preclinical students, and the results were consistent other studies that were conducted in Turkey, China, Saudi Arabia, Italy, and Nigeria [18,30,43,44,45,46]. The lower knowledge scores among undergraduate students highlight the need to refine the current dental curriculum, including infectious diseases epidemiology and control practices. This formal training of the preclinical students will help in increasing their understanding of the safety protocols. Students in the health sciences disciplines should acquire appropriate practical skills for infection control practices. Continuous education and assessment in the clinical setting may aid in improving the learning outcomes [47]. During the COVID-19 pandemic, the lack of practical training among students emerged as a concern, which can be addressed through new technological innovations [46,47,48,49,50]. Educators need to be trained for adopting new virtual platforms of teaching, which could be used in the future crisis. In addition, dental operatories need to be observed for the safety of patients as well as dental professionals [46,47,48,49,50]. In addition, future studies can be conducted to investigate the adherence of healthcare professionals with the recommended infection control practices.

### Study Limitations

This investigation has some limitations. First, by the cross-sectional nature of this study, cause–effect relationships have not been studied. Second, this study’s nonprobability sampling method may have introduced a selection bias as participants were approached via web-based platforms. Third, the sample was not nationally representative, and it was recruited from only the Northern region, limiting our ability to extrapolate our findings to the other dental institutions. Fourth, this study only assessed the general protective behaviors that were related to COVID-19, and it did not include any question related to the adoption of precautionary behaviors in dental clinics. Last, this study may encounter some degree of selection bias due to its cross-sectional design. Prospective studies can be designed to measure safety compliance in the dental practice to dental practice guidelines.

## 5. Conclusions

Our study indicated that dental students had sufficient knowledge regarding the COVID 19 pandemic. The students displayed responsible social behavior, which could be correlated to their level of knowledge on the pandemic. This study highlights the need to restructure the educational curriculum to prepare students to handle COVID-19 and future pandemics. This is critical in improving their knowledge of infection control practices to minimize the risk of nosocomial infections.

## Figures and Tables

**Table 1:** Demographic characteristics of the study population (n = 381).

			Knowledge (Range: 0–100%)		Preventive Behaviors (Range: 0–100%)		Risk Perception (Range: 1–5)	
Variables	Characteristic	n (%)	M ± SD	*p*-value	M ± SD	*p*-value	M ± SD	*p*-value
Gender	Male	79 (20.7)	13.91 ± 0.78	0.04 *	8.75 ± 0.55	0.27 **	2.48 ± 1.1	0.01 *
	Female	268 (70.3)	13.66 ± 0.94		8.82 ± 0.47		2.90 ± 1.0	
	Not reported	34 (9)	-	-	-	-	-	-
Degree course	Undergraduate dental	320 (84)	13.68 ± 0.91	0.2 *	8.79 ± 0.50	0.8 *	2.7 ± 1.0	<0.001 *
	Post-graduate dental	33 (8.7)	13.88 ± 0.90		8.81 ± 0.54		3.6 ± 1.0	
	Not reported	28 (7.3)						
Received formal education about COVID-19	Yes	247 (64.8)	13.70 ± 0.87	0.1 *	8.83 ± 0.50	0.09 *	2.7 ± 1.0	0.02*
	No	102 (26.8)	13.69 ± 1.03		8.73 ± 0.51		3.0 ± 1.0	
	Not reported	32 (8.4)						
Source of education	Social media	193 (50.7)	13.72 ± 1.0	0.2 **	8.78 ± 0.52	0.4 **	2.7 ± 1.0	0.5 **
	TV/Radio	62 (16.3)	13.69 ± 1.0	-	8.82 ± 0.45	-	2.6 ± 1.0	
	College	36 (9.4)	13.94 ± 0.9	-	8.83 ± 0.51	-	3.2 ± 1.1	
	Newspapers	33 (8.7)	13.57 ± 0.75	-	8.76 ± 0.50	-	3.0 ± 1.0	
	Others (Family/Friends)	29 (7.6)	13.43 ± 0.91	-	8.83 ± 0.40	-	2.8 ± 1.0	
	Not reported	28 (7.3)						

* Independent-samples-*t*-test. ** Analysis of variance.

**Table 2:** Assessment of knowledge related to COVID-19 (n = 381).

Items (True or False); Possible Range: (0.0–100.0%)	Correct Answer Rate (Range 0–100%)
COVID-19 is a respiratory infection caused by a new species of coronavirus family. (T)	86.6
2. The first case of COVID-19 was diagnosed in Wuhan, China. (T)	91.3
3. The origin of COVID-19 is not clear, but it seems that it has been transmitted to humans by seafoods, snakes, or bats. (T)	83.2
4. Its common symptoms are fever, cough, and shortness of breath, but nausea and diarrhea were reported rarely. (T)	87.6
5. Its incubation period is up to 14 days with a mean of 5 days. (T)	88.7
6. It can be diagnosed by PCR test on samples collected from nasopharyngeal and oropharyngeal discharge or from sputum and bronchial washing. (T)	86.6
7. It is transmitted through respiratory droplets such as cough and sneeze. (T)	89.8
8. It is transmitted through close contacts with an infected case (especially in family, crowded places and health centers). (T)	90.8
9. The disease can be prevented through handwashing and personal hygiene. (T)	88.7
10. A medical mask is useful to prevent the spread of respiratory droplets during coughing. (T)	89.5
11. The disease can be prevented through no close contacts such as handshakes or kissing, not attending meetings and frequent hand disinfection. (T)	89.2
12. All people in society should wear masks. (T)	87.4
13. Only during intubation, suction, bronchoscopy and cardiopulmonary resuscitation, you have to wear N95 mask. (T)	50.4
14. The disease can be treated by usual antiviral drugs. (F)	45.4
15. If symptoms appear within 14 days from direct contact with a suspected case, the person should inquire at a nearby public health center. (T)	89.2
**Total**	**83.0**

**Table 3:** Preventive behaviors and risk perception of COVID-19 (n = 381).

Items: Preventive Behaviors for COVID-19	Correct Answer Rate (Range 0–100%)
I canceled or postponed meetings with friends, eating out, and sport Events	89.5
I reduced the use of public transportation	90.8
I went shopping less frequently	90.8
I reduced the use of closed spaces, such as library and theatre	90.8
I avoided coughing around people as much as possible	89.2
I avoided places where many people gathered	90.0
I increased the frequency of cleaning and disinfecting items that can be easily touched with hands (i.e., door handles and surfaces)	86.3
I washed the hands more often than usual	89.5
I discussed, with my family and friends, what we should do if infected with COVID-19	82.9
**Total**	**79.9**
**Risk Perception of COVID-19 (Possible Range: 1–5)**	**M ± SD**
I may be infected with COVID-19 more easily than others	2.44 ± 1.25
I am afraid to be infected with COVID-19.	3.11 ± 1.30
**Total**	**2.78 ± 1.05**

M, mean, SD, Standard deviation, COVID-19, Coronavirus disease-2019.

**Table 4:** Correlation between COVID-19 knowledge, protective behaviors, risk perception and age (n = 381).

Variables	Knowledge	Preventive Behaviors	Risk Perception	Age in Years
Knowledge	1.00	-	-	-
Preventive behaviors	0.18 **	1.00	-	-
Risk perception	0.10 *	−0.03	1.00	-
Age in years	−0.08	0.03	0.27 *	1.00

* *p* < 0.05; ** *p* < 0.01.

## Data Availability

Data can be available from corresponding author upon request.

## Abbreviations

COVID-19	Coronavirus disease-19
WHO	World Health Organization
PPE	Personal Protective Equipment
DCI	Dental Council of India
UDMRI	Uttaranchal Dental and Medical Research Institute
PIS	Participant Information Sheet
MERS	Middle East Respiratory Syndrome
SARS	Severe Acute Respiratory Syndrome
M	Mean
S.D	Standard Deviation
